# Vapor Cannabis Exposure Promotes Genetic Plasticity in the Rat Hypothalamus

**DOI:** 10.1038/s41598-019-53516-4

**Published:** 2019-11-14

**Authors:** Julianna N. Brutman, Shuwen Zhang, Pique Choi, Yangzi Zhang, Meagan J. Stotts, Jennifer Michal, Zhihua Jiang, Jon F. Davis

**Affiliations:** 1 Department of Integrative Physiology and Neuroscience, Pullman, USA; 2 Department of Animal Sciences, Washington State University, Pullman, WA USA

**Keywords:** Behavioural genetics, Gene expression profiling, Hypothalamus

## Abstract

It is well established that cannabis use promotes appetite. However, how cannabis interacts with the brain’s appetite center, the hypothalamus, to stimulate feeding behavior is unknown. A growing body of evidence indicates that the hypothalamic transcriptome programs energy balance. Here, we tested the hypothesis that cannabis targets alternative polyadenylation (APA) sites within hypothalamic transcripts to regulate transcriptomic function. To do this, we used a novel cannabis vapor exposure model to characterize feeding in adult male Long Evans rats and aligned this behavioral response with APA events using a Whole Transcriptome Termini Sequencing (WTTS-Seq) approach as well as functional RNA abundance measurements with real-time quantitative polymerase chain reactions. We found that vapor cannabis exposure promoted food intake in free-feeding and behaviorally sated rats, validating the appetite stimulating properties of cannabis. Our WTTS-Seq analysis mapped 59 unique cannabis-induced hypothalamic APAs that occurred primarily within exons on transcripts that regulate synaptic function, excitatory synaptic transmission, and dopamine signaling. Importantly, APA insertions regulated RNA abundance of *Slc6a3*, the dopamine transporter, suggesting a novel genetic link for cannabis regulation of brain monoamine function. Collectively, these novel data indicate that a single cannabis exposure rapidly targets a key RNA processing mechanism linked to brain transcriptome function.

## Introduction

Cannabis is the most widely used illicit substance worldwide^1^ and its recent legalization has brought into question both its therapeutic potential and abuse liability^2–6^. The appetite stimulatory effects of cannabis are well established^7–9^ and have been harnessed to combat anorexia in some patient populations^10–13^. Thus, a better understanding the biological mechanisms that regulate cannabis-induced feeding is necessary to improve treatment options for patients suffering from inappetence. In this context, the hypothalamus is the central nervous system (CNS) region that integrates metabolic information with internal need to regulate appetite and energy balance^14,15^. Within the hypothalamus, genetic programming events regulate food intake and energy balance^16–20^. Separate from genetic imprinting, dynamic changes in mRNA expression patterns within hypothalamic neurons are recognized as powerful processes that regulate food intake and body weight^16–24^. Recent work indicates that the transcriptional reorganization of gene networks offers a means to direct information flows from the genome to behavioral feeding phenotypes^24,25^.

Specifically, new evidence indicates that alternative polyadenylation (APA) of mRNAs, an RNA processing mechanism that generates 3′ termini on transcripts, is a key genetic mechanism that regulates mRNA abundance and localization, and as a result, the biological function of neurons^22,24,25^. Accordingly, this improved understanding of APA biology has led to its use as a biomarker for behavioral disorders and a measure of effectiveness for pharmacotherapies used to mitigate these conditions^26–31^.

In the present study, we hypothesized that cannabis exposure would induce rapid changes in APA to regulate hypothalamic transcriptome function. To address this question, we used a novel plant matter vapor cannabis delivery system in tandem with a Whole Transcriptome Termini Site Sequencing (WTTS-Seq) approach to profile dynamic transcriptional changes within the hypothalamus in male Long-Evans rats. We incorporated assessment of homeostatic and hedonic appetite, analysis of differentially expressed APA events, protein-protein interaction networks, and RNA abundance measures to create an integrative genomics model for cannabis exposure in the hypothalamus. Our results indicate that vapor cannabis exposure promotes increased feeding behavior in the free feeding condition and following behavioral satiation.

In a separate study, we determined that vapor cannabis exposure reduced APA events on transcripts that regulate synaptic function and excitatory synaptic transmission within the hypothalamus. Notably, this observation occurred 1 hr following cannabis exposure, a timepoint aligned with feeding behavior in our model. Biological replication studies indicated that cannabis-induced APA events were functional to alter RNA abundance of Slc6a3, the rat dopamine transporter. In combination, these data highlight APA as a genetic mechanism with rapid restructuring properties that contributes to hypothalamic transcriptome function following cannabis exposure.

## Results

### Cannabis increases food intake in free-feeding rats

**Figure 1:**
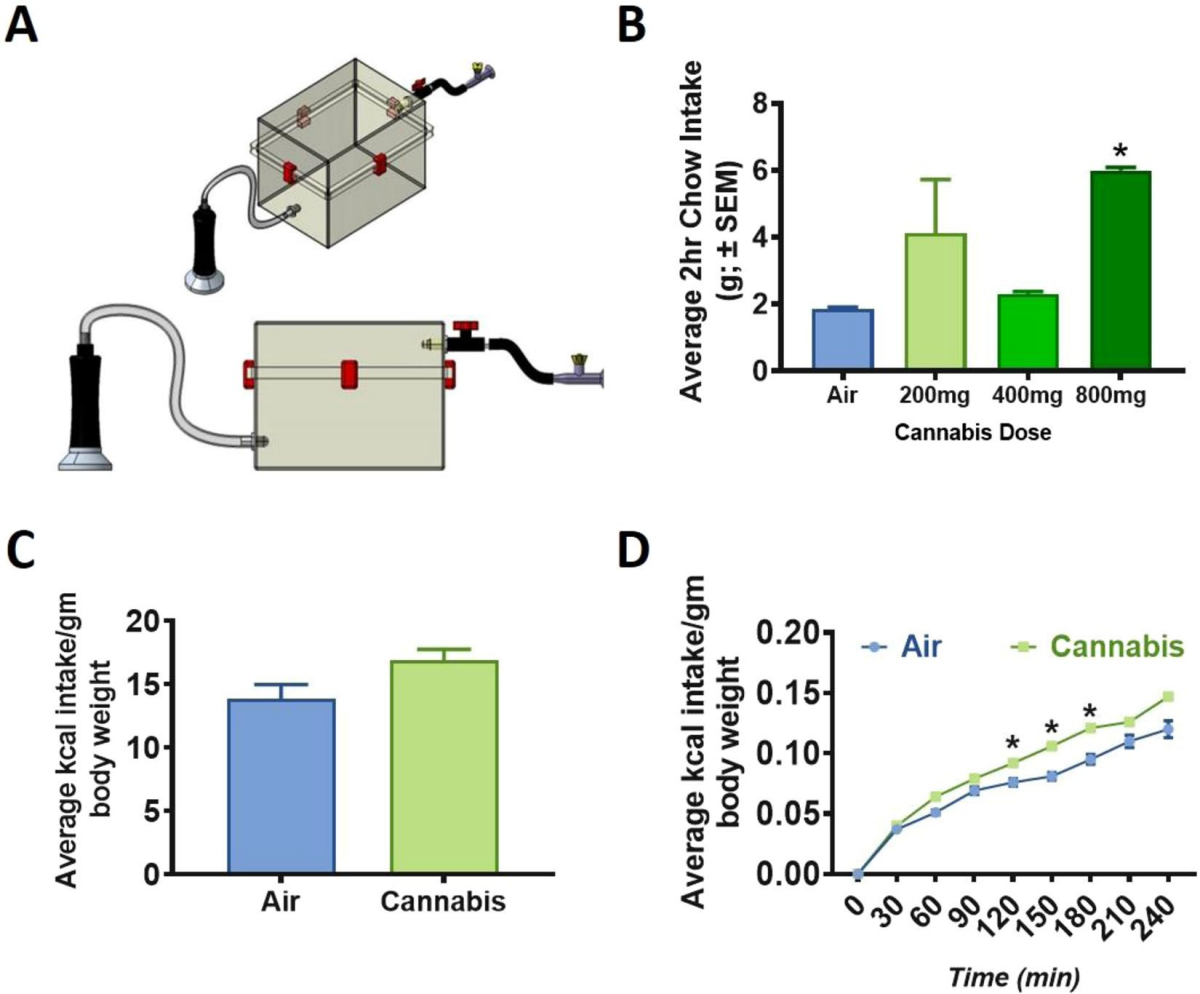
Cannabis Vapor Chamber Design, Feeding Dose Response, and Enhanced Hedonic Feeding. (**A**) Vaporized cannabis was delivered to a positive ventilated rat chamber (Allentown, box dimensions 14 in x 10 in x 7 in; lid dimensions 14 in x 10 in x 2.5 in) via plastic tubing affixed to the VapirRise 2.0 (Vapir, CA) with a plastic 3D-printed union and the cage with a custom designed port. A fan on the vaporizer drives aerosolized cannabis into the chamber during testing at a flow rate of less than 1 L/min. Vaporized cannabis is exhausted with a house vacuum system and chambers were thoroughly cleaned with 70% ethanol between sessions. A plastic stopcock controls the flux of air into and out of the chamber. (**B**) A one-way ANOVA indicated that free-feeding rats receiving 800 mg of cannabis vapor displayed increased feeding behavior (F_1,14_ = 12.6511, p = 0.0032). In contrast, food intake in rats that received 200 or 400 mg of cannabis vapor did not differ significantly from air-exposed controls. (**C**) 2 hr re-feeding on chow prior to air or cannabis exposure did not differ between groups. (**D**) Following behavioral satiation, cannabis exposure led to significantly increased cumulative consumption of Ensure indicating that this manipulation augmented hedonic feeding behavior. Ensure intake in cannabis exposed rats was significantly different from air-exposed rats at 120 min (F_1;14_ = 4.642, p = 0.0491), 150 min (F_1;14_ = 8.0542, p = 0.0132), and 180 min (F_1;14_ = 5.6319, p = 0.0325). There were no significant differences in Ensure intake before or after these time points.

For the present study, we created a new cannabis exposure chamber to conduct the trials (Fig. 1A). We observed that cannabis exposure significantly increased chow intake in free-feeding rats. Specifically, our dose response determined that vaporized exposure to 800 mg of cannabis produced significant increase in standard chow intake (p < 0.05) relative to air-treated controls (Fig. 1B). Furthermore, rats receiving the behaviorally-relevant dose of 800 mg displayed elevated levels of plasma THC at 10 min (35.54 ± 9.51 ng/mL; F_1,6_ = 7.4419, p = 0.0343), 30 min (28.94 ± 5.02 ng/mL; F1,6 = 19.0266, p = 0.0048), and 60 min (21.80 ± 2.75 ng/mL; F_1,6_ = 14.7686, p = 0.0085) in comparison to air-exposed controls that displayed undetectable levels of THC.

### Cannabis exposure augments hedonic food intake

Preclinical work indicates that THC injection enhances the hedonic properties of sucrose in male rats^32^. These findings are consistent with clinical reports indicating that patients using cannabis prefer to eat palatable foods^7,33^. In the present study, vapor cannabis exposure induced significant feeding from a palatable liquid diet in sated rats (p < 0.05; Fig. 1C,D). As per previous reports utilizing the dessert effect paradigm, total chow intake was negligible and did not differ between control and experimental rats over the 4 hr time course of Ensure access^34,35^. Importantly, hedonic intake of Ensure was significantly increased following vapor cannabis exposure, an effect that emerged 120 min following exposure and persisted for 60 min. Collectively, these data indicate that vapor cannabis exposure stimulates feeding in the presence or absence of caloric need.

### Cannabis-induced differentially expressed APAs at conventional and non-conventional RNA sites

**Figure 2:**
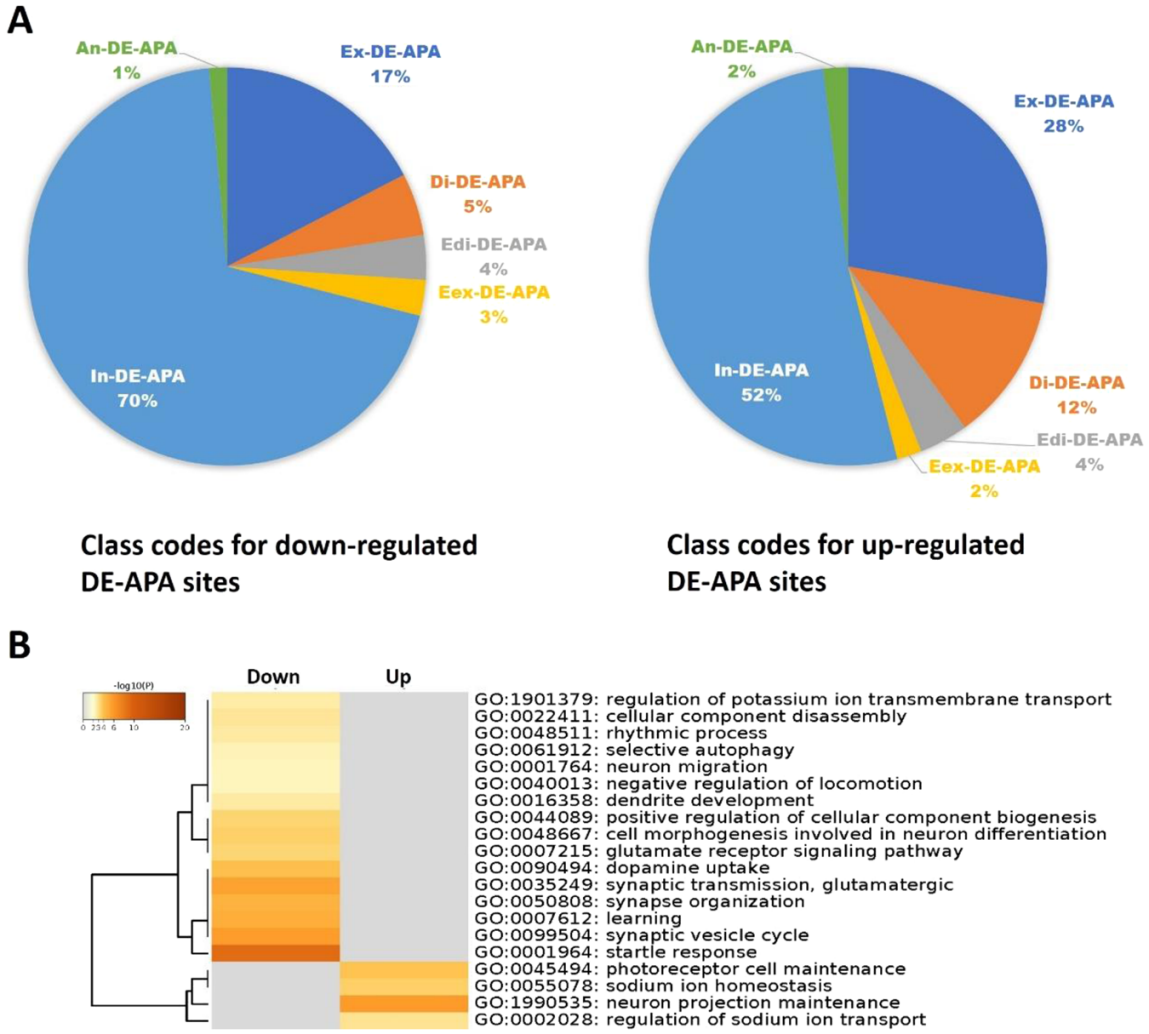
Characterization of down- and up-regulated DE-APA sites induced by cannabis exposure. (**A**) Within-gene locations defined are further classified into two groups: conventional APA sites located in exonic (Ex-DE-APA), distal (Di-DE-APA) or extended distal (Edi-DE-APA) regions; and non-conventional APA sites derived from extended exonic (Eex-DE-APA), intronic (In-DE-APA) and antisense (An-DE-APA) regions. Interestingly, these two groups of DE-APA sites were distributed differently (P = 0.0188). (**B**) DE-APA genes associated with 138 down- and 50 up-regulated DE-APA sites were used to enrich pathways using the Metascape program^8^. Our functional ontology revealed that cannabis exposure impacted DE-APAs on gene networks implicated in (**1**) learning impairment, (**2**) memory, (**3**) mood, (**4**) movement, (**5**) sensory perception, and (**6**) cognition.

Using 16 reads per site as a cutoff, we identified a total of 98,356 APA sites expressed in the hypothalamus (Table S1). Among them, 64,313 APA sites were assigned to the currently annotated genes in rat. With P < 0.01, 309 differentially expressed APA (DE-APA) sites were identified, including 243 down-, but only 66 up-regulated in rats with cannabis exposure as compared to air-treated controls (Table S2). Among them, 138 down- and 50 up-regulated DE-APA sites are associated with genes. The Cuffcompare program (version 2.2.1) classified these sites into six types: exonic (Ex-, confined in exonic regions), distal (Di-, transcript ends), extended distal (Edi-, located within 2 kb downstream of reference transcripts), extended exonic (Eex-, extended from exonic regions to intronic regions with at least 10 bp), intronic (In-, completed in the intronic regions), and antisense APA (An-DE-APA, exonic regions, but with opposite direction) sites, respectively. While the former three types are considered as the conventional APA sites, the latter three are considered non-conventional APAs and thus their usage frequencies significantly differed between down- and up-regulated DE-APA sites (P = 0.0188). As shown in Fig. 2A, the down-regulated DE-APA sites favored more intronic sites (increased by 18%), but less exonic sites (decreased by 11%) and sites (decreased by 7%) than the up-regulated DE-APA sites. Using “GO Biological Processes” and all DE genes expressing these DE-APA sites, the Metascape program enriched 16 down- and 4 up-regulated pathways induced by cannabis exposure (Fig. 2B).

### Cannabis exposure rapidly reduced APA events on hypothalamic transcripts

**Figure 3:**
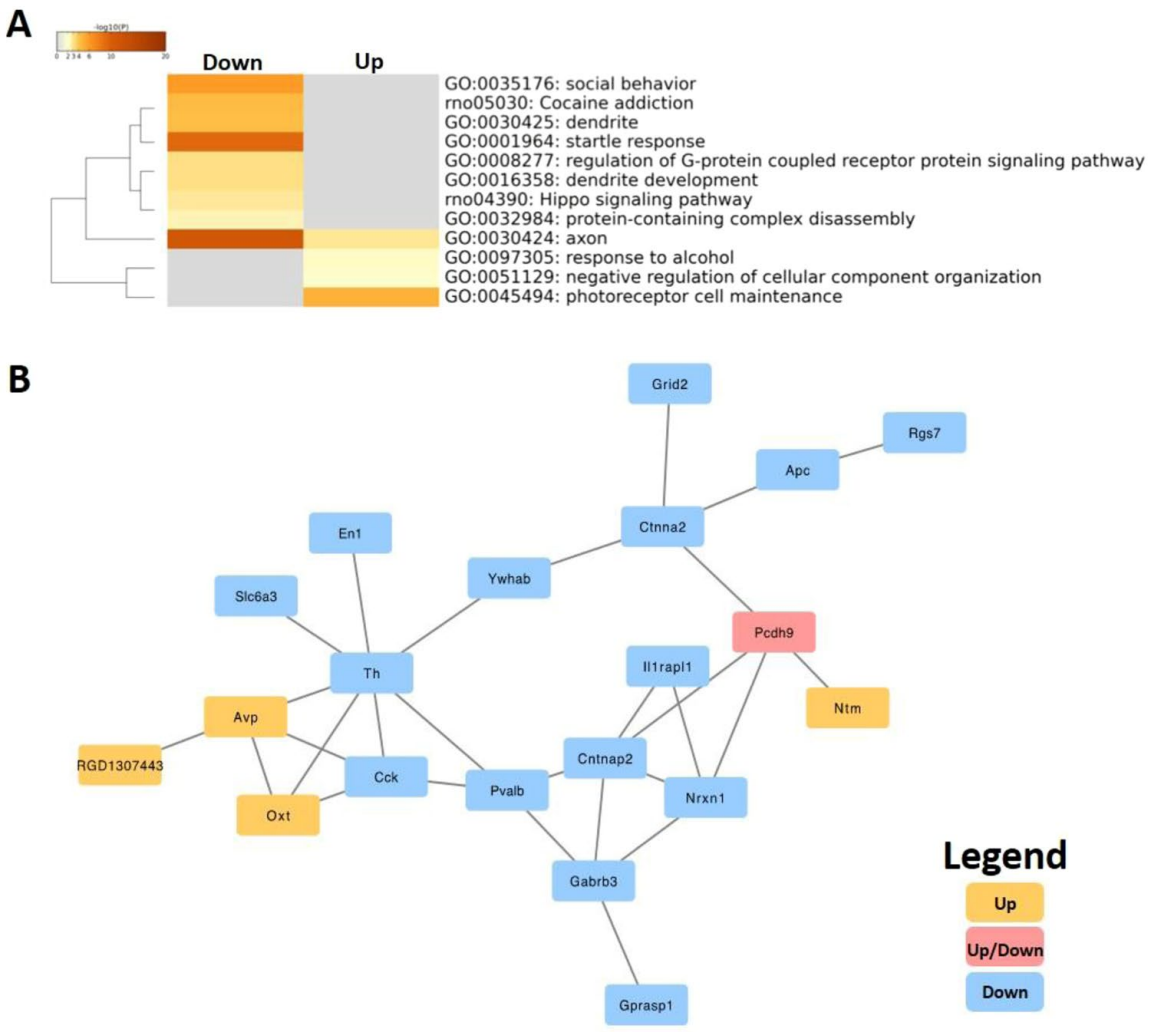
Gene pathway enrichment analysis for DE-APA protein coding genes and protein-protein interaction network of DE-APA related genes. (**A**) We identified 24 differentially expressed APAs exclusively up-regulated and 35 differentially expressed APAs lost or down-regulated in acute cannabis exposed rats. Terms with p-value (<0.01) were defined as significant pathways. Using this criteria cannabis exposed rats had two main pathway clusters down-regulated: (**1**) behavioral response and (**2**) synaptic function. (**B**) Yellow nodes represent transcripts with increased APA events, blue nodes represent gene transcripts with decreased APA events in cannabis exposed rats relative to air controls. The pink nodes indicate APA impacted gene transcripts with both up- and down- regulated pathway enrichment in response to cannabis exposure. Our functional protein-protein interaction (PPI) network mainly demonstrate enrichment of pathways from transcripts with decreased APA events, while we observed less pathway enrichment from gene transcripts with increased APA events.

Vapor cannabis exposure rapidly altered APA on a collection of hypothalamic mRNA transcripts whose functional proteins broadly regulate synaptic function. Specifically, after filtration and *in silico* validation to confirm all valid differentially expressed APAs, 59 differentially expressed APAs (p < 0.01) were present in cannabis-exposed rats relative to air controls (Table 1). Based on log2 fold changes, vaporized cannabis stimulated increased APA on 24 differentially expressed APAs and in contrast 35 differentially expressed APAs were down-regulated. Two main pathway clusters further defined differentially expressed APAs induced by cannabis exposure: **1)** behavioral response and **2)** synaptic function (Fig. 3A).

**Table 1:** Table of the 59 most significantly up- and down-regulated differentially expressed APA sequences as determined by the Metscape analysis.

Gene Symbol	Biotype	Class Code	Average Reads Air Control	Average Reads CPM Exposed	p-Value
*Slc6a3*	mRNA	Intronic	290.75	37.25	4.01E-11
*En1*	mRNA	Distal	33.5	4.5	9.67E-08
*LOC108352127*	lncRNA	Distal	25.75	2	2.11E-06
*Pcdh9*	mRNA	Intronic	13.25	69.75	4.04E-06
*Th*	mRNA	Distal	323.75	139.5	7.27E-05
*P3h3*	mRNA	Distal	90.5	25.25	0.000111599
*Il1rapl1*	mRNA	Intronic	11	0.5	0.000187807
*Cck*	mRNA	Distal	126.25	59	0.000222467
*Ptms*	mRNA	Extended Exonic	700.5	331	0.00026513
*Chgb*	mRNA	Exonic	37.25	3.25	0.000389995
*Zfp717*	mRNA	Exonic	0.5	9.75	0.000581046
*Ccsap*	mRNA	Exonic	8.25	0.75	0.000678713
*Nrxn1*	mRNA	Intronic	45.5	13.5	0.000815366
*Tmem130*	mRNA	Exonic	3.5	19	0.000995052
*Grid2*	mRNA	Intronic	8.75	0.5	0.001282604
*Avp*	mRNA	Distal	2006.5	5559.75	0.001406465
*Ntm*	mRNA	Exonic	1.25	14.25	0.001458359
*Pvalb*	mRNA	Exonic	121.25	50.5	0.002598358
*Gprasp1*	mRNA	Exonic	10	1	0.002712193
*Ywhab*	mRNA	Extended Exonic	15.25	3.75	0.002865265
*Otud7b*	mRNA	Extended Exonic	5.75	0.25	0.002879665
*Ccdc186*	mRNA	Exonic	7	0.5	0.00311328
*Bcat1*	mRNA	Exonic	0.25	9	0.003400789
*Ctnna2*	mRNA	Intronic	4.75	0.25	0.003970525
*Pcdh9*	mRNA	Intronic	14	3.75	0.00435816
*Dctn1*	mRNA	Extended Exonic	5.25	28.5	0.00473107
*Mir1188*	miRNA	Extended Distal	5.75	0	0.004929709
*Pacsin1*	mRNA	Exonic	9.25	0.5	0.005254815
*Kif5a*	mRNA	Exonic	11.25	56.75	0.005269956
*Iqcb1*	mRNA	Intronic	0	4	0.005422377
*Lmo4*	mRNA	Exonic	1.75	12.5	0.005556857
*Ythdc1*	mRNA	Intronic	1	7.5	0.00575421
*Zfp407*	mRNA	Intronic	11.25	32.75	0.005813125
*LOC103690249*	lncRNA	Intronic	1.75	11	0.005940773
*Gabrb3*	mRNA	Intronic	4.25	0.25	0.005987257
*Arhgef9*	mRNA	Intronic	0	6	0.006142502
*Prkg1*	mRNA	Intronic	7.75	1	0.006209398
*Apc*	mRNA	Exonic	70	30.75	0.006312224
*Wipi2*	mRNA	Extended Distal	9	2.25	0.006429699
*Cntnap2*	mRNA	Intronic	7	0.25	0.006439209
*Drosha*	mRNA	Intronic	5.5	17.75	0.007340514
*Nbas*	mRNA	Intronic	9.75	28.75	0.007352915
*Patz1*	mRNA	Distal	16.75	41.5	0.007354928
*Rgs7*	mRNA	Intronic	7	0.75	0.007609581
*Zfp106*	mRNA	Exonic	13.5	1.75	0.00770108
*Cdh23*	mRNA	Exonic	1.75	9.75	0.007732982
*Ralgps1*	mRNA	Intronic	4	0	0.007786372
*Bsg*	mRNA	Distal	268.75	627	0.007791359
*Grin2d*	mRNA	Exonic	4.75	0	0.007994193
*Oxt*	mRNA	Distal	695.75	1446.75	0.008433266
*Zc3h13*	mRNA	Intronic	9.25	2.5	0.008545125
*Zfp106*	mRNA	Exonic	4.75	0	0.008732914
*LOC102555913*	lncRNA	Extended Distal	10.75	3.5	0.009126488
*RGD1307443*	mRNA	Exonic	8.5	25.75	0.009276461
*Map1a*	mRNA	Exonic	5	31	0.0094434
*Nrxn1*	mRNA	Exonic	3.75	0.25	0.009472235
*LOC686774*	mRNA	Extended Exonic	7.75	0.5	0.009647936
*Eif2d*	mRNA	Intronic	3.5	13.25	0.00979683
*Med13l*	mRNA	Exonic	21.5	57.5	0.00994487

Gene name, biotype, class code, and average reads between cannabis treatment and air treated controls are provided with p-value statistics.

### Predicted protein-protein interactions (PPI) from cannabis-induced differentially expressed APAs

The analysis of our functional PPI map of differentially expressed APA related transcripts revealed a primary enrichment of a rat protein “interactome” implicated in synaptic function (Fig. 3B). Notably, most of the functional pathways (15/24) were enriched for down-regulated APA following cannabis exposure. RNA transcripts implicated in the down-regulatory network included G-Protein coupled receptor (GPCR) transcripts such as Rgs7, Ywhab, and Gprasp1. In addition, we detected glutamatergic and GABAergic transcripts such as Grin2d, Grid2, and Gabrb3 as well as dopamine-related transcripts including TH and Slc6a3. We also detected transcripts implicated in learning and synaptic development that included Cck and En1. Pcdh9 was the only protein identified with both up and down regulated differentially expressed APAs (Fig. 3B).

### Acute cannabis exposure impacted gene expression on identified DE-APA transcripts

**Figure 4:**
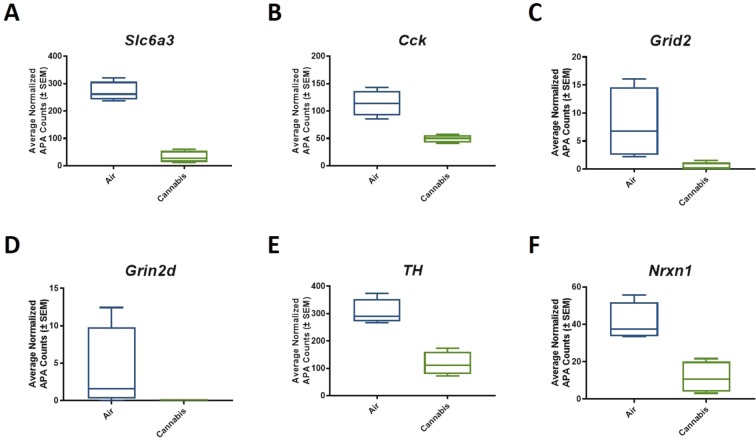
Significant pathways from the functional ontology were selected to have closer inspection of genes associated with vaporized cannabis treatment. This analysis revealed that vaporized cannabis exposure selectively reduced APA events (p-value = p < 0.01) on mRNA transcripts that regulate glutamate transmission such as (**A**) *Slc6a3*, (**B**) *Cck*, (**C**) *Grid2*, (**D**) *Grin2d*, (**E**) *TH*, and (**F**) *Nrxn1*.

**Figure 5:**
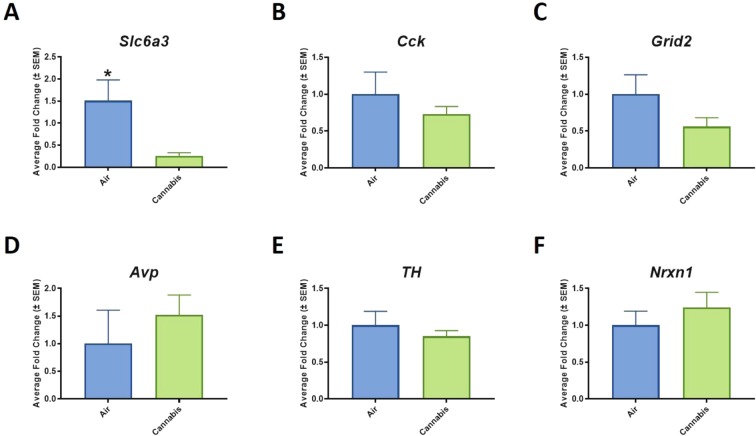
Reduction of APA events and mRNA abundance in cannabis exposed rats. Specifically, reduced APA led to down-regulation of (**A**) Slc6a3, (**B**) *Cck*, (**C**) *Grid2*, and (**E**) *TH*. Although all were reduced relative to air-treated controls, only *Slc6a3* reached statistical significance (F_1;14_ = 5.9377, p = 0.0299). In contrast, (**D**) *Avp* abundance increased relative to air treated controls, and (**F**) *Nrxn1* did not differ from air-treated controls.

Within the 35 differentially expressed APAs that were down regulated, cannabis exposure reduced APA insertions on mRNAs that regulate glutamate neurotransmission, dopamine signaling, and synapse formation including: *Grid2, Slc6a3, TH, Nrxn1*, and *Pcdh9* (p < 0.01) (Fig. 4A–F). We next independently validated mRNA abundance to corroborate the functional relevance of reduced APA among these transcripts. Results indicate that attenuated APA events secondary to cannabis exposure led to reduced abundance of *Slc6a3* (p < 0.05; two-tailed t-test) and *Grid2* mRNA, however, *Grid2* did not reach statistical significance. The abundance of *TH, Nrxn1*, and *Pcdh9* were unaltered relative to air-treated controls (Fig. 5A–F).

## Discussion

The goal of the present study was to determine how vapor cannabis exposure influences transcriptome function in a brain region that regulates feeding behavior. From these efforts, several significant findings emerged. First, our data indicate that cannabis can promote feeding in the presence or absence of a caloric need, a phenomenon that occurred over a short duration of time following a single exposure to cannabis. Second, we aligned this behavioral response with differentially expressed APA events, functional RNA abundance, and PPI in the hypothalamus, thereby informing novel post-transcriptional mechanisms that may contribute to cannabis induced feeding behavior. In this regard, cannabis reduced APA on mRNA transcripts implicated in excitatory synapse function and dopaminergic signaling. Collectively, these data indicate that pulmonary inhalation of the cannabis plant alters the genetic landscape of appetite regulatory brain regions.

Our novel system represents one of a limited number of attempts^36,37^ to design a preclinical rodent model with high face validity for vaporization of the cannabis plant. Cannabis contains hundreds of cannabinoids – offering many possible effects beyond those observed from THC, or CBG, phytocannabinoid derivatives that stimulate appetite in patients and rodents^38–42^. To this point, previous studies have indicated that botanical drug substances have greater benefits than the individual extracted cannabinoids^43–45^, highlighting the potential value of whole plant matter cannabis for appetite stimulation. In this context, pulmonary inhalation is the most widely used route of administration by cannabis users^46–51^. In the present study, we detected elevated plasma THC levels in cannabis exposed rats 10 min after vapor exposure. Importantly, cannabis exposure increased both homeostatic food intake in free feeding rats and hedonic food intake in behaviorally sated rats. The nature of feeding behavior following vapor cannabis exposure included a delayed onset and short duration, effects supported by previous studies using injection of phytocannabinoid derivatives^52,53^. Thus, consistent with previous reports, a delayed onset of appetite and short-term duration of feeding are common features to both phytocannabinoid and vapor cannabis-induced appetite.

Our next goal involved understanding how a behaviorally characterized dose of cannabis altered the genetic landscape within the hypothalamus. The functional ontology analysis we performed revealed that cannabis exposure selectively reduced APA on mRNAs as opposed to other biotypes. We detected APAs within multiple regions of affected transcripts following cannabis exposure. For instance, cannabis exposure resulted in an equal amount of intronic APA sites in transcripts that were up (35%) or down-regulated (35%). However, more extended distal APA sites (p sites, by 3%) and more extended exonic APA sites (e sites, by 9%), but less exonic APA sites (c sites, by 8%) and less distal sites (o sites, by 4%) were triggered by cannabis exposure. It has been proposed that use of distal Poly A sites preferentially place longer transcripts under negative regulatory control by miRNAs, whereas the loss of distal APAs spares transcripts from miRNA regulation^54^. Our data show that novel use of genome sequences (extended exonic APA sites and extended distal sites) was reduced by cannabis exposure. One interpretation of these results is that acute cannabis exposure protects hypothalamic mRNA transcripts from miRNA regulation by reducing distal APA events, a contention that requires further experimental validation by artificially manipulating APA events.

The majority of APA events on protein-coding mRNAs occurred on transcripts responsible for regulating synaptic transmission. Specifically, we observed a significant decrease of APA events on multiple transcripts implicated in dopaminergic neurotransmission, such as *Slc6a3*, the dopamine transporter, which is tightly linked to addiction vulnerability and feeding behavior^55–59^ and on tyrosine hydroxylase (TH), the rate limiting enzyme for dopamine production^60^. This observation becomes relevant when considering that acute THC exposure promotes dopamine efflux in the brains of humans and rodents^61–65^. Our data highlight cannabis-induced APA as a novel genetic mechanism that potentially regulates CNS dopamine function. Vapor cannabis exposure also decreased APA events on *Grid2* and *Grin2d*, ionotropic glutamate receptors that regulate excitatory synaptic transmission^66,67^. Interestingly, we detected decreased APA events on *Nrxn1*, a transcript involved with synaptic plasticity^68–70^ and clinical nicotine dependence^71^. Future studies that focus on manipulating APA sites will determine the functional relevance of this process for overall cellular function and *in vivo* behavior.

In terms of functional relevance of APA events, cannabis exposure led to significant down-regulation of *Slc6a3* and decreased RNA abundance for all transcripts except *Grid2d* (not measured) and *Nrxn1*, which increased relative to air controls however these measures did not reach statistical significance. These data suggest that APA events are sometimes coupled with mRNA abundance changes at an acute timepoint following cannabis exposure. In this regard, it is possible that monitoring mRNA abundance and/or stability at later time points following cannabis treatment may have yielded tighter coupling to APA events. These contentions notwithstanding, our PPI analysis uncovered a protein interactome where TH, Ctnna2, and Nrxn1 served as hubs between APA identified transcripts involved in dopamine signaling, cell adhesion, and synapse formation, respectively.

The present findings align with previous reports detailing the effects of exogenous THC on the brain and may offer a further integration of the genetic mechanisms that influence cannabinoid effects on neurobiological function. For example, work from Miller *et al*. reported that adolescent THC exposure stimulated gene expression changes in transcripts such as *Pcdhac1* and *Slc441a*, synaptic transcripts that code for proteins with highly similar functions to those identified by our APA analysis^72^. Further work from the same group examining transgenerational THC exposure in the ventral and dorsal striatum identified genetic expression differences in *Grin1*, *Grin2A*, *Grin2B*, *Gria1*, and *Gria2*^73^, one of the first studies to show that exogenous THC exposure modifies glutamatergic signaling. In that study, THC induced genetic expression differences in the brain by differential methylation in the nucleus accumbens, a pre-transcriptional regulator of gene expression^73^. The data we present here suggest that cannabis targets APA as a mechanism to regulate glutamatergic transcripts following transcription and that this process is rapid. Similar to functional expression changes brought about by methylation, our analyses identified post-transcriptional differentially expressed APA events as a possible regulator of mRNA abundance. Our APA data become particularly intriguing when considering prior preclinical work that report endogenous surges of glutamate in the arcuate nucleus (ARC) as mice consume palatable food^74,75^. Moreover, a newly recognized population of dopamine neurons in the ARC has received attention for their ability to stimulate food intake^76–78^. When viewed collectively, these data support the notion that the pathway targets of cannabis may be biologically conserved across brain regions, whereas the genetic regulation of each pathway takes multiple forms.

In conclusion, our studies demonstrate that pulmonary inhalation of cannabis vapor is sufficient to promote appetite. Cannabis-induced feeding was defined by a delayed onset and short duration. A behaviorally relevant dose of cannabis led to decreased APA in the hypothalamus, reflected in a functional decrease in key protein-coding RNA transcripts linked to dopaminergic neurotransmission. Collectively, our data highlight the ability of vapor cannabis exposure to rapidly target an RNA processing mechanism linked to regulation of brain transcriptome function.

## Methods and Materials

### Animals

Male Long-Evans rats (Harlan, IN) weighing 275–300 g were used as experimental subjects. All rats were maintained on given *ad libitum* chow and water except when indicated. Rats were housed in an environmentally controlled vivarium on a reverse light cycle and acclimated for 1 week prior to experimental manipulations. All work adhered to Institutional Animal Care and Use Committee (IACUC) guidelines at Washington State University (WSU), and all experimental protocols used in the study were approved by the Animal Care and Use Committee at Washington State University (WSU IACUC 6523 and 6527).

### Diets

Rats were maintained *ad libitum* on regular rodent chow (3.41 kcal/g, LabDiet 5001) while in their home cages. Chocolate-flavored Ensure (1.8 kcal/g) was provided to measure hedonic feeding behavior.

### Apparatus

Vapor chambers were constructed by attaching a cannabis vaporizer (VapirRise 2.0, Vapir Inc., Santa Clara, CA) to sealed positive ventilation rodent shoebox cages (Allentown Inc., Allentown, PA) as illustrated in Fig. 1A. The measurements of the Allentown positive ventilation rodent shoebox cages are as follows: base dimensions 14 in × 10 in × 7 in; lid dimensions 14 in × 10 in × 2.5. The flow rate of the VapirRise 2.0 was less than 1 L/min and cannabis plant matter was combusted at 360 °F.

### Cannabis

Dry plant matter *cannabis sativa* (7.8% THC, 0.5% CBD) classified as “bulk marijuana” was obtained from NIDA Drug Supply (Research Triangle, NC). Cannabis consisted of ground whole plant matter, with large stems removed for combustion in the Vaporizer 2.0 apparatus, as described above.

### General procedure

For each experiment, rats (n = 8/group) were habituated to the vapor chambers (Fig. 1) for 10 min across 5 days prior to any experimental manipulation. On test days, the rats were exposed to cannabis (7.8% THC, 0.5% CBD, whole plant matter, NIDA drug supply, Research Triangle, NC) vaporized over a 10 min period. Control rats were placed into an identical apparatus with an unfilled vaporizer and served as “air-treated” controls. Following establishment of the dose-response, plasma was collected to measure THC concentration from a subset (n = 4/group) of rats. For this experiment, plasma THC measures were performed following exposure to a behaviorally relevant cannabis dose (800 mg) that stimulated food intake.

For assessment of hedonic feeding, behaviorally sated rats were given access to chocolate flavored Ensure for 4hrs following air or vapor cannabis exposure. We used the behavioral assessment of hedonic feeding to guide measurement of hypothalamic APA events in a separate cohort of male rats (n = 8/group). For APA analysis, free feeding rats were exposed to cannabis and food was removed for a 1 hr period prior to collection of hypothalamic tissue for WTTS-Seq analysis. A third cohort of rats (n = 8/group) was exposed to an identical cannabis-dosing/food restriction paradigm served as a biological replicate for measurement of hypothalamic RNA abundance.

### Experiment 1: dose determination – cannabis-induced feeding response & plasma THC

To determine a dose-response for food intake, free-feeding male rats (n = 8) were exposed to 200 mg, 400 mg, and 800 mg of vaporized cannabis and chow intake was measured over a 2 hr period. Following behavioral characterization, plasma levels of 11-nor-carboxy-Δ-9-tetrahydrocannbinol (THC), the most abundant metabolite of Δ-9-tetrahydrocannbinol, were analyzed in a separate cohort of male LE rats (n = 4/group) using a Max Signal THC ELISA kit (Bio Scientific #5013, Austin, TX) at 10, 30, and 60 following cannabis exposure. No food was offered during THC measurements.

### Experiment 2: cannabis and hedonic appetite

We used our “dessert effect” paradigm^34,35^ to measure hedonic intake of palatable food or feeding in the absence of caloric need. To accomplish this, rats were nutrient deprived for 20hrs then subsequently resupplied with unlimited access to standard rodent chow for 2hrs to induce behavioral satiation. After behavioral satiation, rats were exposed to 800 mg cannabis then provided with a highly palatable liquid diet (chocolate flavored Ensure). Chow and ensure intake were then measured every 30 min for 4hrs. To reduce neophobia, all rats were exposed to a small amount of Ensure prior to the test. As in previous studies utilizing this dessert effect paradigm, rats require no training to complete this task^21,34,35,79,80^_._

### Experiment 3: hypothalamic APA events following cannabis exposure

In this experiment, APA events were measured in hypothalamic tissue collected 1 hr following vaporized cannabis exposure. Specifically, upon sacrifice, whole brains were flash frozen in 2-methlybutane, and the hypothalamus was micro-dissected out. Total RNA was isolated from hypothalamic samples with TRIzol Reagent (650 μL/sample, Life Technologies Corp., Carlsbad, CA). DNA was removed by treating total RNA with DNase (AM1906, Ambion) to prevent DNA contamination. Total RNA quantity was measured with the Quant-It RiboGreen RNA Assay Kit (Life Technologies Corp., Carlsbad, CA) and quality assessed by fragment analysis (Advanced Analytical Technologies, Inc., Ankeny, IA).

### Wtts-seq library construction

In the present study, we used our WTTS-Seq protocol^23,81^ to construct libraries for all rats. For each individual sample, 2.5 μg of total RNA was chemically fragmented using RNA fragmentation buffer (AM8740, Ambion) as the first step, then enrichment of poly(A) + RNA was accomplished with Dynabeads oligo(T) magnetic beads (61002, Ambion) followed by reverse transcription with SuperScript III Reverse Transcriptase (18080, Invitrogen) to synthesize first strand cDNA. Next, both 5′- and 3′- adaptors were added to fit the Ion Torrent sequencing platform. All RNA molecules were removed by both RNases H (M0297L, NEB) and I (EN0601, Thermo Scientific) to prevent contamination, 250–500 bp first-strand cDNA molecules were selected with solid-phase reversible immobilization beads (A63880, Beckman Coulter), and second-strand cDNA was synthesized by PCR. Lastly, all libraries were sequenced using an Ion PGM™ Sequencer at Washington State University.

### Read filtration, mapping, and APA predication

Sequencing of 8 WTTS-seq libraries yielded a total of 47,803,583 raw reads, ranging from 2,457,707 to 9,828,283 reads per sample. Raw reads with a minimum of 50% bases covered at 90% identity (Q10) were filtered with the FASTX Toolkit (http://hannonlab.cshl.edu/fastx_toolkit/) to produce high-quality reads. According to our WTTS-seq method’s strand-specific feature, in-house Perl scripts were applied to trim all Ts (thymine(s)) at beginning of each read and keep at least 16nt long reads for mapping, which produced 47,678,618 reads and 46,668,933 reads, respectively. The rat reference genome in FASTA format and annotation file in GFF format were downloaded from NCBI (https://www.ncbi.nlm.nih.gov/genome/term = rat) with the version Rattus norvegicus Rnor_6.0 (Annotation Release 106). TMAP (version 3.4.1, https://github.com/iontorrent/TMAP) was chosen as the mapping tools to complete all read mappings. The polyadenylation adjacent site was defined as the first nucleotide of each trimmed read, and then it was clustered with others that are within a 24-nt window. After clustering, all polyadenylation sites were determined as predicted APA sites^23,81^.

### Cannabis-induced APA gene annotation in rats

The Cuffcompare program (version 2.2.1) was used to assign gene information to each predicted APA site, gene biotypes and class codes are also included. Genome level information contained scaffold number, strand direction and 5′- and 3′-end coordinates on each chromosome, while gene level information included ID number, strand, biotype, symbol, and transcript ID for each APA site.

### DE-APA data annotation, pathway enrichment

The DESeq. 2 package (https://bioconductor.org/packages/release/bioc/html/DESeq. 2.html) was used to identify DE- APAs between the cannabis exposed condition and controls. Quality control mapping (MAPQ < 70) was applied to avoid low mapping reads resulting in 123 differentially expressed APAs with relatively low mapping quality that were removed. We selected differentially expressed APAs with significant p-values of 0.05. If the average expression levels of differentially expressed APAs under the cannabis condition were greater than differentially expressed APAs in the control condition, their fold changes were set up as the positive values (the former average divided by the latter average). If differentially expressed APAs were up-regulated in the control condition relative to the cannabis exposed condition, their fold changes were designated as negative values. Both gene sets associated with differentially expressed APAs that were up or down-regulated in cannabis exposed condition were used together as inputs into the Metascape program for both pathways. The UCSC Genome Browser (Kent, W. J. *et al*. 2002, http://genome.ucsc.edu/) and BLAT (Kent, W. J. *et al*. 2002, http://genome.ucsc.edu/) were used *in silico* validation to confirm all valid differentially expressed APAs at the gene level.

### Experiment 4: functional independent validation of APA reads

To independently validate the APA reads, we utilized real time quantitative PCR (RT-qPCR). For RT-qPCR validation, total RNA from cannabis and air treated rats (n = 8/group) was extracted using a Qiagen RNeasy Micro Kit (Qiagen, MD) according to the manufacture’s protocol. Total RNA was quantified using a Nanodrop 2000c spectrophotometer, and a High Capacity cDNA Reverse Transcription Kit with RNase Inhibitor (Applied Biosystems, CA) was utilized to reverse transcribe complementary DNA (cDNA). Following a 15-fold dilution with DEPC water, template cDNA was mixed with Fast SYBR® Green Master Mix (Applied Biosystems, CA) and primers, and then assayed in triplicates on a 96 well plate with appropriate negative controls to detect contamination. RNA expression was measured with a ViiA 7 RT-qPCR system (Life Technologies Corporation, NY) for Rn45s (housekeeping gene), Slc6a3, Grid2, TH, Nrxn1, and Pcdh9. The 1:100 diluted primers (IDT, San Diego, CA, USA) were as follows: *Rn45s*, left-GTGGAGCGATTTGTCTGGTT and right-CGCTGAGCCAGTTCAGTGTA; *Slc6a*, left- TGTGAGGCATCTGTGTGGAT and right-GGTGGTGATGATTGCGTCTC; *Grid2*, left- TCTTGCCGCTTTCCTCACTA and right-CTGCGGAATCCAAGACTGTG; *Nrxn1*, left- GTCCGAGATGTCCACCTCAA and right-ATCCGTGGTCTGGCTGATAG; *TH*, left- CAGTACAAGCACGGTGAACC and right-TAGCATAGAGGCCCTTCAGC and *Pcdh9*, left- TCTCAGCGTCGTGTTACCTT and right-GGTGTGAGATCAGGGTTCCA. A melt-curve analysis was included on all qPCR assays to ensure primer specificity. A 2^ΔCt^ method was utilized for relative quantification of target transcript, and results were normalized to controls.

### Statistical analysis

Food intake was analyzed by univariate analysis of variance (ANOVA). All statistical comparisons were conducted at 0.05α level. The DESEq. 2 package in R was used to normalize raw reads among samples, separate transcriptome profiles within and between treatment groups and to identify differentially expressed APAs with Padj (corrected) <0.1. All APAs, including the differentially expressed APAs were further annotated using the HOMER (v4.9) software suite (http://biowhat.ucsd.edu/homer/) to determine mapping locations, including TTS (transcription terminal site, by default defined from −100 bp to +1 kb), exons, introns, 3′UTR, 5′UTR, TSS (transcription start site, by default defined from −1 kb to +100 bp) and intergenic regions. The protein coding genes associated with the differentially expressed APAs were then used as input in the Metascape program and pathways were enriched using “GO Biological Processes”^8^. Protein-protein interaction networks were identified using the STRING database (https://string-db.org/)^37^ and visualized using the graphical network Cytoscape software (version 3.5.1.). The χ^2^ test was used to detect significant differences among or between categories using R. In addition, the raw WTTS-Seq reads have been submitted to The NCBI Gene Expression Omnibus (GEO) (http://www.ncbi.nlm.nih.gov/geo/) under accession no GSE100349.

### Accession codes

WTTS-Seq data for this study can be found at the NCBI Gene Expression Omnibus (GEO) (http://www.ncbi.nlm.nih.gov/geo/) under accession code no GSE100349

## Supplementary information

Dataset 1

Dataset 2
